# Patient-Reported Outcomes in Intraoral Bone Block Augmentation Compared to GBR Procedures Prior to Implant Placement: A Systematic Review

**DOI:** 10.3390/jcm14155331

**Published:** 2025-07-28

**Authors:** Sepehr Salahi, Mohamad Kamal Shaar, Jeremy Pitman, Stijn Vervaeke, Jan Cosyn, Faris Younes, Thomas De Bruyckere

**Affiliations:** Department of Periodontology and Oral Implantology, Faculty of Medicine and Health Sciences Oral Health Sciences, Ghent University, C. Heymanslaan 10, B-9000 Ghent, Belgium; m.shaar@ugent.be (M.K.S.); jeremy.pitman@ugent.be (J.P.); stijn.vervaeke@ugent.be (S.V.); jan.cosyn@ugent.be (J.C.); faris.younes@ugent.be (F.Y.); thomas.debruyckere@ugent.be (T.D.B.)

**Keywords:** postoperative complications, intraoperative complications, alveolar ridge augmentation, dental implants

## Abstract

**Objective**: To compare the effect of different bone augmentation procedures, namely, autogenous bone blocks (ABBs) versus guided bone regeneration (GBR), on patient-reported outcomes (PROMs). **Methods**: This systematic review was conducted according to the PRISMA guidelines. A MEDLINE, Embase, and Web of Science search was conducted by two independent reviewers in combination with a free-hand search in relevant journals until June 2025. Outcomes were PROMs to enhance our understanding of the evolution of these procedures. **Results**: The electronic search yielded 6291 articles. After title screening, 67 articles were further analyzed for abstract review, which resulted in 14 articles eligible for full-text reading. Six articles were finally included based on the exclusion and inclusion criteria with a total of 295 patients. The overall study quality was low, since only two RCTs could be included. One study demonstrated a high risk of bias. Different PROMs were examined and compared such as pain, edema, neurosensory disturbance, Patient-Reported Predominant Symptom, OHIP-14, postoperative analgesic usage, willingness to repeat, and likelihood to recommend. Meta-analysis was not achievable due to a lack of direct comparisons and heterogeneity in terms of PROMs. Evaluation points varied between pretreatment and up to nearly 10-years of follow-up. **Conclusions**: Despite significant heterogeneity and reporting, this systematic review concluded that ABB and GBR are well-tolerated procedures. Trends such as transient postoperative pain and swelling with a minor occurring of neurosensory disturbances were reported in a few studies. Overall, a good perception of postoperative recovery was reported for both treatment modalities. Good quality of life was noted related to GBR procedures. Patient-reported outcomes were only analyzed for patients who completed the entire follow-up period. This may introduce bias, as patients who dropped out and were more likely to experience complications were not represented, potentially resulting in a more favorable portrayal of the outcomes. Further well-conducted prospective studies with a long follow-up are needed for an evidence-based evaluation and comparison of PROMs for these procedures.

## 1. Introduction

Dimensional changes of the alveolar bone are a common phenomenon occurring after tooth loss. The alveolar bone is a tooth-supporting structure. Without the presence of teeth, three-dimensional changes in both horizontal and vertical dimensions occur, which may impede the proper placement of implants. During 3 to 6 months, horizontal and vertical bone loss predominates at a rapid reduction [1]. In the field of dental implantology, an optimal bone volume and quality is crucial for the long-term success of dental implants [2,3]. A recent ITI consensus report indicated that implants placed with a thin buccal wall are associated with less favorable clinical outcomes [4]. Subsequently, according to the current literature, about 69.7% of non-molar sites need an additional bone grafting procedure prior to implant placement compared to 45.9% of molar sites [5].

Several augmentation procedures are available for the reconstruction of the alveolar crest to provide sufficient bone to place dental implants [6]. Inlay or onlay techniques can be used to achieve sufficient bone volume for future implant placement. However, inlay bone techniques are mostly used and known as an adequate method of increasing bone volume in cases with sufficient amount of horizontal bone volume [7]. Autogenous bone blocks (ABBs) and guided bone regeneration (GBR) with the use of autogenous bone particles are a widely used approach for the reconstruction of the jaw [8]. Currently, autogenous bone is still regarded as the golden standard for augmentation procedures due to their osteoinductive, osteoconductive, and osteogenic properties [8,9,10,11]. The most common intraoral sites for retrieval of autogenous bone are the symphysial region and ramus of the mandible [12]. However, harvesting autogenous bone requires an additional surgical intervention, which introduces an additional risk of post-surgical complications and donor site morbidity. This in turn is an extra challenge for the patients and can be seen as a disadvantage [13,14].

Guided bone regeneration (GBR) refers to the usage of a membrane to exclude non-osteogenic tissues from interfering with bone regeneration [15]. Nevertheless, GBR procedures may entail the exposure of the membrane, potentially leading to postoperative complications [16]. In 2021, Javier Sánchez-Sánchez et al. [17] published a systematic review comparing ABBs with GBR. The ABB technique utilizes autologous bone blocks for augmentation, whereas the GBR technique employs membranes to stabilize and contain the included biomaterials. Even though the different treatment methods, they concluded that both techniques were found to be effective in terms of clinical bone gain and horizontal alveolar ridge gain. Complications were also reported on infections and temporary paresthesia. These were only reported by clinicians, but no reporting was found on patient-reported outcome measures (PROMs). PROMs are defined as any report of the status of a patient’s health condition that comes directly from the patient, without interpretation of the patient’s response by a clinician or others [18]. In the field of dentistry, PROMs are an essential part of assessing the success of a dental treatment and essential to give the appropriate to the patients’ needs [19]. In particular, this review will focus on the onlay approach within the augmentation procedures as the inlay grafting requires a different surgical protocol making a direct comparison in terms of PROMs less meaningful. There is a scarcity of studies specifically reporting on PROMs as a primary outcome. To the best of our knowledge, this is the first systematic review to specifically investigate PROMs between ABBs and GBR, given that PROMs help enhancing patient engagement and improve the patient’s outcomes [20].

## 2. Materials and Methods

The following analysis was performed according to the guidelines of the Cochrane Collaboration and the principles of the PRISMA-guidelines (Preferred Reporting Items for Systematic Reviews and Meta-Analyses) statement for a systematic review [21]. The review was registered in December 2023 in the International Prospective Register of Systematic Reviews (PROSPERO) at the UK’s National Institute for Health Research (NIHR), University of York, Centre for Reviews and Dissemination with registration number: CRD42023488704.

### 2.1. Objectives

The primary objective of this study was to compare PROMs between ABBs and GBR. The focused research question was: Do patients undergoing intraoral bone block augmentation, compared to guided bone regeneration, differ in pain and other PROMs?

By utilizing PICO elements, we derive the following components:
PatientSystemically healthy adult patient with alveolar ridge deficiencies;InterventionBone block augmentation using bone from the retromolar area (ABB);ComparisonGuided bone regeneration (GBR);OutcomePROMs such as pain, edema, hematoma, neurosensory disturbance, willingness to repeat, OHIP-14.

### 2.2. Eligibility Criteria

Inclusion was designed according to PICO guidelines found in Table A1 in the following Appendix A.

Article selection was performed according to the following inclusion criteria:

- Human clinical studies published in English or articles when a direct translation was possible;
- Intraoral bone grafts;
- At least 18-years old patients;
- Systemically healthy patients;
- Patients with alveolar ridge deficiency;
- Studies reporting on bone augmentation;
- Data on at least one outcome variable of interest.

Studies were excluded based on study design (cross-sectional studies, letters to editors, reviews) or whether the studies reported on

- Extraoral bone grafts;
- Sinus floor elevation procedures;
- Bone ring techniques;
- Distraction osteogenesis;
- Alveolar ridge splitting;
- Full edentulous patients;
- Patients taking anti-resorptive drugs;
- Patients who underwent radiation therapy;
- Patients with pathologies affecting bone metabolism (i.e., osteoporosis, osteopenia, rheumatoid arthritis);
- Containing insufficient information on the surgical protocol and bone augmentation procedures.

### 2.3. Information Sources and Search Strategy

The intended information sources consisted of a comprehensive electronic search of MEDLINE, Embase, and Web of Science databases by two independent reviewers (SS and MS) ranging from 2007 until 2024. Two reviewers (SS and MS) performed this search, initially focusing on the removal of duplicates. Subsequently, the same reviewers screened the articles by title and abstract. If doubt arose at either the title or abstract screening level, the article would be further evaluated at the next level to avoid overlooking appropriate studies. For articles with no abstract available, a full-text analysis was resolved by a third reviewer (TDB). The detailed description of the different searches in described in Table A2. The usage of ‘OR’ and ‘PICO’ parameters were combined to become a final search query. To assess the inter-rater reliability in the selection of eligible studies, the kappa-coefficient was calculated at both the title, abstract, and full-text level. Additionally, a manual search was conducted until June 2025 in the following journals and beforementioned databases: Journal of Clinical Periodontology, International Journal of Oral and Maxillofacial Surgery, Journal of Periodontology, and British Journal of Oral and Maxillofacial Surgery.

### 2.4. Data Extraction

Information pertaining to study design, follow-up duration, patient demographics, and treatment outcomes was extracted from the included articles. In instances where multiple follow-up periods were available, preference was given to the study with the longest follow-up. Information about treatment modalities was requested by email from the corresponding authors in case of missing or unclear data. If unanswered, the studies were excluded.

### 2.5. Risk of Bias Quality Assessment

Included articles were assessed on their quality by two independent reviewers (SS and MS) using the revised Cochrane Risk-of-Bias Tool (RoB2) for randomized trials as developed by Sterne et al. [22]. During this procedure, the following criteria were scored: (1) bias arising from the randomization process, (2) bias due to deviations from intended interventions, (3) bias due to missing outcome data, (4) bias in measurement of the outcome, and (5) bias selection of the reported results. All 5 domains were judged as low risk of bias, some concerns, or high risk of bias. The Newcastle Ottawa Scale, as developed by Wells et al. [23], was used to assess the quality of the non-randomized studies according to the following criteria: (1) selection of the study group; (2) comparability of study groups; (3) outcome assessment or ascertainment of exposure. The included non-randomized studies were categorized as poor, fair, or good quality based on the number of stars assigned to each domain.

### 2.6. Data Analysis

The data were primarily analyzed from a qualitative perspective. Due to heterogeneity in study design, surgical techniques, evaluation time points, and outcome measures, quantitative synthesis was not feasible. The analysis was therefore limited to the descriptive reporting of patient-reported outcomes (PROMs), including pain, swelling, neurosensory disturbances, analgesic usage, and OHIP-14. Mean values and standard deviations were reported where available. Given the variability in clinical protocols and PROMs assessment across studies, neither a pairwise nor a network meta-analysis could be conducted.

## 3. Results

### 3.1. Search

**Figure 1:**
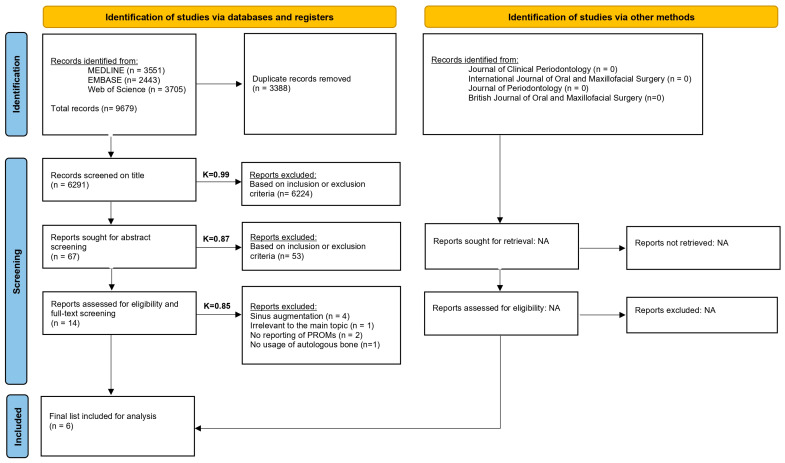
PRISMA flowchart.

The detailed search strategy is shown in Figure 1. A comprehensive electronic search conducted in MEDLINE, Embase, and Web of Science yielded a total of 9679 records. Gray literature did not lead to additional articles, and no further articles were found by a manual search. After removal of duplicates, 6291 articles were included for title screening. After title screening, 67 records were included for an abstract screening. The inter-rater agreement on title, abstract, and full-text screening were, respectively, 0.99 (*p* < 0.001), 0.87 (*p* < 0.001), and 0.85 (*p* < 0.001). After abstract screening, 14 articles were included for a full-text screening analysis. Eight articles were excluded after full text analysis; reasons for their exclusion can be found in Supplementary Table S1 [24,25,26,27,28,29,30,31]. Thus, six articles met the inclusion criteria for final analysis [32,33,34,35,36,37]. Information about treatment modalities were requested by email from the corresponding authors in case of unclear data missing or if unanswered, the studies were excluded. A response rate of 100% was seen.

### 3.2. Description of the Selected Studies

Table 1 presents the characteristics of the included studies [32,33,34,35,36,37], published between 2016 and 2025. Two RCTs and four observational studies were included. Bone graft interventions were performed prior to implant placement. This descriptive analysis comprised a total of 295 patients with a mean ranging from 36.3 to 71.1 years. A total drop-out rate of 5.8% was seen. Augmentation procedures in the included studies were performed at sites such as the maxilla, the mandible, or both. A total of 355 bone graft procedures were performed with a total of 420 implants prior to bone augmentation. The study of Korsch et al. [33] did not specify the number of implants placed in the augmented sites. Evaluation time points varied across the studies from pretreatment to a mean follow-up term of almost 10 years. The PROMs showed variability in the included studies. Outcomes such as pain, swelling, neurosensory disturbance, patient-reported predominant symptom, postoperative analgesic usage, and willingness to repeat were reported. One article also used the OHIP-14 questionnaire as an assessment method. A detailed description of tools used in the corresponding included articles can be found in Supplementary Table S2.

### 3.3. Risk of Bias Assessment

Table 2 presents the risk of bias assessment of all included RCTs. None of the studies demonstrated an overall low risk of bias. Of the two studies included in this analysis, the study of Shiezadeh et al. [36] was judged to have a high overall risk of bias. This was due to deviations from the intended intervention, as implant placement in the second surgery could not be performed: one graft was lost after wound dehiscence and graft loss, while the other became infected. The study of Korsch et al. [33] was assessed as having some concerns, primarily due to issues to the randomization process. Table 3 and Table 4 illustrates the risk of bias assessment of all non-randomized studies using the Newcastle Ottawa Scale (NOS) for observational studies. Two studies were designed as cohort studies, one as a case series study. The cohort studies [32,34] were rated as good quality, although some concerns were noted due to lack of non-exposure group. The case–control study of Thoma et al. [37] was rated good quality. The presence and the proper selection of a non-exposure group was a notable aspect of this study. Three out of four studies were given a score between 7 and 8, demonstrating good overall quality. Only one study was reported to have poor quality. This was due to the absence of a non-exposed group and the inadequate reporting of follow-up periods.

**Table 1:** Study characteristics.

Author	Study Design	Follow-Up Points	No. of Patients	No. of Dropouts	No. of Bone Grafts	No. of Implants	Mean Age (y)	Type of Graft	Augmentation Site	Test Group	Control Group	Outcome Variables
Bayram et al., 2024 [32]	rCT	Week 1 1 year	70	0	70	70	44.96	Autograft	Mandible or Maxilla	ABB	NR	Neurosensory disturbances
Shiezadeh et al., 2023 [36]	RCT	26 Weeks	25	2	13	37	36.27–36.5	Autograft Vs. Xenograft	Mandible or Maxilla	ABB (Collagen Membrane)	GBR (Collagen membrane)	Neurosensory disturbances in donor sites (ABB only) and recipient sites (ABB and GBR) swelling
Thoma et al., 2019 [37]	rCC	Mean follow-up of 10 years	38	0	19	67	51.9–71.1	Autograft	Mandible or Maxilla	ABB with Bio-Oss© and Bio-Guide©	NB	Sensory problems Willingness to repeat the procedure Likelihood to recommend the procedure
Lorenz et al., 2025 [34]	PCT	Pretreatment Weeks 1, 3, 12, and 24 Time of implant placement Definitive prosthesis 6 months 8 months 1 year	46	8	119	91	50	Autograft and Xenograft	Mandible	GBR	NR	Patient-reported pain perceptions using VAS and OHIP-14
Sakkas et al., 2016 [35]	rCS	NR	86	0	104	155	37.9	Autograft	Mandible or Maxilla	ABB with Bio-Guide©	NR	Neurosensory disturbance
Korsch et al., 2014 [33]	RCT	Day 1, 3, 7, 14, and 28	30	0	30	NR	47.9–52.1	Autograft and Xenograft	Maxilla	ABB	GBR	Pain and swelling in the donor and recipient sites, postoperative analgesic usage and patient-reported predominant symptom

rCT: retrospective cohort study; RCT: randomized controlled trial; rCC: retrospective case–control; PCT: prospective cohort study; rCS: retrospective case series; NB: native bone; ABB: autologous bone block; GBR: guided bone regeneration; NR: not reported.

**Table 2:** Revised Cochrane Risk-of-Bias Tool for Randomized Trials (RoB2). Icons indicate a low risk of bias (☑), some concerns (☐), and a high risk of bias (☒) in all five domains. The last column illustrates the overall risk of bias of included RCTs.

	D1	D2	D3	D4	D5	Overall
Shiezadeh et al., 2014 [36]	☑	☒	☑	☑	☐	☒
Korsch et al., 2014 [33]	☐	☑	☑	☑	☑	☐

**Table 3:** Newcastle Ottawa Scale for Observational Studies—cohort study.

Author	Selection				Comparability	Outcome			Total	Overall Quality
	Representatives of the exposed cohort/case (Maximum: ✸)	Selection of the non-exposed cohort/case (Maximum: ✸)	Ascertainment of exposure (Maximum: ✸)	Demonstration that outcome of interest was not present at the start of the study (Maximum: ✸)	Compare ability of cohorts on the basis of the design or analysis (Maximum: ✸✸)	Assessment of outcome (Maximum: ✸)	Was follow-up long enough for outcomes to occur (Maximum: ✸)	Adequacy of follow-up cohorts (Maximum: ✸)		
Lorenz et al., 2024 [34]	✸		✸	✸	✸	✸	✸	✸	7	Good
Bayram et al., 2024 [32]	✸		✸	✸	✸✸	✸	✸	✸	8	Good
Sakkas et al., 2016 [35]	✸		✸	✸		✸		✸	5	Poor

✸ Star Allocation: A study can receive a maximum of one star for each numbered item within the Selection and Outcome categories. Comparability can receive a maximum of two stars.

**Table 4:** Newcastle Ottawa Scale for Observational Studies—case–control study.

**Author**	**Selection**				**Comparability**	**Outcome**			**Total**	**Overall Quality**
	Is the case definition adequate? (Maximum: ✸)	Representativeness of the cases? (Maximum: ✸)	Selection of controls (Maximum: ✸)	Definition of controls (Maximum: ✸)	Comparability of cases and controls on the basis of the design or analysis (Maximum: ✸✸)	Ascertainment of exposure (Maximum: ✸)	Same method of ascertainment for cases and controls (Maximum: ✸)	Non-response rate (Maximum: ✸)		
Thoma et al., 2024 [37]	✸	✸	✸	✸	✸	✸	✸	✸	8	Good

✸ Star Allocation: A study can receive a maximum of one star for each numbered item within the Selection and Outcome categories. Comparability can receive a maximum of two stars.

### 3.4. Outcomes

The overall description of results can be found in Table 5.

#### 3.4.1. Pain

The assessment of pain reported by the patients varied across the included studies. Two out of six studies [33,34] reported on the patient’s pain perception using the Visual Analog Scale (VAS). These studies evaluated pain intensity, duration, and evolution. The RCT by Korsch et al. [33] compared ABB with GBR. In this study, pain decreased from day 1 (D1) to day 28 (D28) at the donor site in patients undergoing ABB. However, at the recipient site, an initial increase of pain was noted from day 1 (D1) until day 3 (D3), followed by a decrease from day 3 (D3) to day 28 (D28). In the GBR group, pain levels remained stable during the first 3 days and then decreased significantly from day 3 (D3) to day 28 (D28) at both donor and recipient sites. While patients in the ABB group reported less pain at the recipient site than those in the GBR group from day 1 (D1) to day 28 (D28), the difference was not statistically significant. The open, prospective, single-cohort, multicenter clinical study of Lorenz et al. [34] included patients with ridge defects that required GBR before implant placement. A gradual reduction in pain was observed from week 1 (W1) (3.1 ± 2.3) to week 24 (W24) (0.3 ± 1.2) with pain levels remaining low from week 24 (W24) (0.1 ± 0.7) to month 8 (M8) (0.2 ± 0.7).

#### 3.4.2. Swelling

Two studies reported about patients’ perception of swelling [33,36]. Korsch et al. [33] reported a decrease in swelling at the donor site in the ABB group from day 1 (D1) until day 28 (D28). At the recipient site, a slight increase in postoperative swelling was noted until day 3 (D3), followed by a decrease until day 28 (D28). A similar pattern was observed in the GBR group, where swelling increased slightly from day 1 (D1) to day 3 (D3) and then decreased until day 28 (D28) at both donor and recipient sites. When comparing ABB and GBR groups, swelling was reported to be higher at the donor site and lower at the recipient site in the ABB group although the differences were not statically significant. Shiezadeh et al. [36] assessed swelling over a two-week follow-up period in both ABB and GBR groups, observing that swelling gradually subsided during that time.

#### 3.4.3. Neurosensory Disturbance

Four studies reported patient-reported discomfort related to neurosensory disturbances following augmentation procedures [32,35,36,37]. Bayram et al. [32] noted that eight patients experienced a present neurosensory disturbance in the first postoperative week. However, after 1 year, no patients reported any such disturbances. Shiezadeh et al. [36] reported that none of the patients experienced neurosensory disturbances. The retrospective case series of Sakkas et al. [35] highlighted 11 patients who suffered from a temporary hypoesthesia of the mental area and 3 patients who reported neurosensory disturbances of the tongue. At the time of implant placement, none of these patients presented with a neurosensory disturbance. The study of Thoma et al. [37] used a questionnaire to assess neurosensory disturbances. Seven patients in the augmentation group presented with an extraoral skin sensitivity at the donor site after the primary augmentation, while three patients presented with intraoral sensitivity at the donor site. The majority of the observed complications were not present anymore at the follow-up examination.

**Table 5:** Study outcomes.

Author	Treatments		Pain			Swelling		Neurosensory Disturbance			Patient-Reported Predominant Symptom (Pain/Swelling)		Postoperative Analgesic Usage	OHIP-14 (Quality of Life)	Willingness to Repeat	Likelihood to Recommend
			D	R	NS	D	R	D	R	NS	Ds	Rs				
Bayram et al., 2024 [32]	ABB		NR	NR	NR	NR	NR	NR		W1–Y1: Decrease	NR	NR	NR	NR	NR	NR
Shiezadeh et al., 2023 [36]	ABB	GBR	NR	NR	NR	NR	Swelling until 4 weeks FU	0/11	0/11	NR	NR	NR	NR	NR	NR	NR
Thoma et al., 2019 [37]	ABB	NB	NR	NR	NR	NR	NR	EO: 7/19 IO: 3/19	0/19	NR	NR	NR	NR	NR	Median 10 (AB range 5.5–10) (NB range 7–10)	Median 10
Lorenz et al., 2025 [34]	GBR		NR	NR	W1–W24: Decrease W24–M8: Stable	NR	NR	NR	NR	NR	NR	NR	NR	Pre-W1: Increase W1–Y1: Decrease	NR	NR
Sakkas et al., 2016 [35]	ABB		NR	NR	NR	NR	NR	NR	NR	11/104 (10.4%)	NR	NR	NR	NR	NR	NR
Korsch et al., 2014 [33]	ABB		D1–D28: Decrease	D1–D3: Increase D3–D28: Decrease	NR	D1–D28: Decrease	D1–D3: Increase D3–D28: Decrease	NR	NR	NR	D1–D7: More swelling than pain	D1–D7: More swelling than pain	D1–D11: Decrease After D11: usage = 0	NR	NR	NR
	GBR		D1–D3: Stable D3–D28: Decrease	D1–D3: Stable D3–D28: Decrease	NR	D1–D3: Increase D3–D28: Decrease	D1–D3: Increase D3–D28: Decrease	NR	NR	NR	D3: More swelling than pain	D1–D7: More swelling than pain	D1–D2: Increase D2–D11: Decrease After D11: usage = 0	NR	NR	NR

ABB: Autologous Bone Block; GBR: Guided Bone Regeneration; D: Day; Ds: Donor Site; Rs: Recipient Site; NS: Not-Specified; NB: Native Bone; NR: Not-Reported.

#### 3.4.4. Patient-Reported Predominant Symptom

One study compared pain and swelling throughout the follow-up period [33]. The ABB procedures presented with greater swelling than pain in both the donor and recipient sites at the 1-week (W1) follow-up. With regard to the GBR-procedures, swelling was more pronounced on day 3 (D3) at the donor site, while the recipient site showed a similar pattern as for the ABB procedures.

#### 3.4.5. Postoperative Analgesic Usage

The dosage of analgesic medication used postoperatively reported by the patients was only reported in a single study [33]. In the study, patients were instructed to use ibuprofen 400 mg as an analgesic, with a maximum daily dosage of three tablets. In the ABB group, a decrease in analgesic usage was observed from the 1st to the 11th postoperative day. However, in the GBR group, an increase in analgesic usage occurred from day 1 (D1) to day 2 (D2), followed by a gradual decrease until the 11th postoperative day. Thereafter, no patients in either group reported using analgesics, with no significant difference between the treatment modalities as a result. The patients in the ABB group required a total of 45 tablets, whereas the patients in the GBR group required 42 tablets.

#### 3.4.6. OHIP-14

Use of the OHIP-14 questionnaire was only reported in one study [34], and consisted of a 4-point questionnaire with a total range between 0 and 56 to document the patients’ perceived quality of life. Assessments were made at six different time points throughout the study, ranging from pretreatment up to 1-year follow-up. Results showed a slight increase in scores during the first week after the surgery followed by gradual decrease until the end of the 1-year follow-up. it was also noted that the OHIP-14 score returned to the pretreatment level by week 12 (W12) postoperatively. These findings indicated that GBR had a minimal impact on the quality of life. Additionally, patients presenting with a lack of keratinized mucosa showed a reduction in quality of life (*p* = 0.022). A sub-analysis was also conducted by the authors to determine the impact of wound dehiscence on the patient-reported outcome of quality of life. Results showed that OHIP-14 scores were higher in the dehiscence group than the non-dehiscence group.

#### 3.4.7. Willingness to Repeat and Likelihood to Recommend

One study reported on the willingness to repeat and the likelihood to recommend the procedure [37], using a Visual Analog Scale (VAS). Three questions were used as evaluation points: ‘How would you rate the procedure?’; ‘Would you repeat the procedure?’; and ‘Would you recommend the procedure?’ The median VAS-score for rating the surgical procedure was slightly lower in the GBR group compared to the native bone group. For the last two questions, both treatment modalities reported a median VAS score of 10. However, the ABB group had a lower minimum score of 5.5 compared to 7 in the native bone group (See Table 5). 

## 4. Discussion

The present systematic review investigated the reporting of different PROMs in autologous bone block augmentation procedures compared with widely used GBR techniques. In this study, six studies were included out of 6291 publications that met the inclusion criteria and were selected for a detailed analysis [32,33,34,35,36,37]. Due to the extreme heterogeneity in terms of study design, surgical protocol methods, evaluation time points, and quantification of outcome variables, a descriptive analysis was performed. The analysis was performed on a total of 295 patients who had been treated by either GBR or autologous bone grafted from the retromolar ramus area or symphysial region. In studies where the site of autologous bone was not specified, the authors were contacted via email for further clarification. This review was performed for one reason, namely, the low overall specific description of PROMs with a low standardized cataloging and therefore being the first systematic review specifically reporting on these topics.

### 4.1. Neurosensory Disturbance

Neurosensory disturbance was the most frequently reported complication in the included studies. Four out of six studies reported neurosensory disturbances following augmentation procedures [32,35,36,37]. The studies highlight the variability of neurosensory outcomes associated with such procedures. The difference in reported outcomes may be attributed to variation in surgical techniques and the clinical expertise of the operator. Piezoelectric surgery may appear to offer advantages over conventional surgical rotary instruments in terms of safety for the alveolar nerve and soft tissues [38,39]. Two of the four studies concluded that the neurosensory disturbances were temporary and resolved over time [32,35]. This finding is consistent with the study of Rodrigo Santos Pereira et al. [40], a prospective investigation evaluating neurosensory disturbances after autogenous bone graft harvesting from the retromolar area or mental area. However, it is important to note that harvesting autologous bone from the mentum (chin) region carries a higher risk of transient or permanent neurosensory disturbance of the alveolar nerve region [41,42]. Quantitative methods are recommended for the assessment and quantification of alveolar nerve disturbances and to allow for more insight into the prevention and communication with the patient [43]. The usage of local buccal and oral infiltration can be used to warn the surgeons in case of reaching the alveolar nerve [44]. Further research is needed since no reporting was found in the GBR groups.

### 4.2. OHIP-14

Only one study reported OHIP-14 scores specifically related to GBR procedures indicating a gradual improvement in patients’ quality of life over time. Notably, higher OHIP-14 scores were observed in the group experiencing wound dehiscence compared to non-dehiscence group. This difference may be attributed to the negative impact of wound dehiscence, which has been shown to adversely affect health-related quality of life as well as social functioning and mental health [45]. Wound dehiscence is a well-documented risk factor for GBR failure, primarily due to its association with secondary infections, which may contribute to elevated OHIP-14 scores [46].

### 4.3. Pain and Swelling

The prevalence of pain and swelling, as self-reported by patients, was evaluated in two of the six included studies for pain and in two studies for swelling. Korsch et al. [33] reported postoperative pain and swelling at both donor and recipient sites. To allow for consistent patient-reported VAS (Visual Analog Scale) scoring, the donor site was always the retromolar region of the mandible, and the recipient site was consistently the anterior maxilla. Korsch et al. [33] also analyzed postoperative analgesic usage and operative time to investigate potential relationships with the severity of patient-reported complaints/discomfort, including pain and other physical or emotional responses. However, since none of these parameters showed statistically significant differences between the groups, no definitive correlation could be established. The study suggests that the comparable pain and swelling levels, as measured by VAS scores, might be due to the fact that postoperative complications are mainly associated with the manipulation of soft tissues. This may explain why reducing the amount of harvested bone did not lead to a noticeable reduction in postoperative discomfort at the donor site. Similarly, at the recipient site, the manipulation of soft tissue, with in mind the periosteal incision required to mobilize and tension-free cover the augmentation may contribute more significantly to postoperative discomfort than the augmentation procedure itself

Several important limitations should be considered when interpreting the findings of this systematic review. Since patient-reported outcomes were only available for patients who completed the entire follow-up period and not for those who discontinued their participation, it is likely that the sample represents a higher risk group for complications. This exclusion introduces a potential selection bias and may result in an overly optimistic portrayal of the outcomes. Firstly, the possible publication and language bias. Secondly, the substantial clinical and methodological heterogeneity across the included studies precluded the possibility of conducting a meta-analysis and quantitative synthesis. This heterogeneity increases the risk of selection bias and hampers direct comparisons between ABB and GBR procedures. Thirdly, as several of the included studies had a retrospective design, the conclusions drawn should be interpreted with some caution due to the inherent risk of bias associated with this type of study. Fourthly, most studies lacked the use of standardized and validated tools for measuring PROMs. Only one study employed the OHIP-14 questionnaire, whereas the others relied primarily on subjective VAS scores with inconsistent timing and interpretation. Lastly, the follow-up periods varied greatly among the studies. Despite these limitations, the current evidence provides a valuable foundation for future research and highlights the importance of integrating PROMs into implant-related studies. To strengthen future conclusions and enhance clinical relevance, well-designed and standardized randomized controlled trials with extended follow-up periods, validated outcome measures, and consistent reporting protocols are strongly encouraged.

## 5. Conclusions

This systematic review demonstrated that, despite variations in methodology and heterogeneity in reporting across the included studies, the effects of ABBs and GBR appear to be well-tolerated procedures, associated with minimal long-term pain, limited neurosensory disturbances, and a generally positive perception of postoperative recovery and quality of life. The overall strength of this review is considered low due to the presence of high risk of bias in some included studies and the scarcity of randomized controlled trials. Patient-reported outcomes were only analyzed for patients who completed the entire follow-up period. This may introduce bias, as patients who dropped out and were more likely to experience complications were not represented, potentially resulting in a more favorable portrayal of the outcomes. Therefore, future RCTs with longer follow-up periods are needed to enable direct comparisons and to provide robust data on PROMs for a better comparison between these treatment modalities, which remain underreported due to the scarcity of available publications.

## Data Availability

The dataset that supports the findings of this study are available from the corresponding author upon reasonable request.

## Supplementary Materials

The following supporting information can be downloaded at: https://www.mdpi.com/article/10.3390/jcm14155331/s1, Table S1: Reasons for exclusion. References [24,25,26,27,28,29,30,31] are cited in the supplementary materials. Table S2: Instruments used for reporting PROMs.

## Abbreviations

The following abbreviations are used in this manuscript:

ABB	Autologous Bone Block
GBR	Guided Bone Regeneration
PROMs	Patient-Reported Outcome Measures
VAS	Visual Analog Scale
OHIP-14	Oral Health Impact Profile-14
RCT	Randomized Controlled Trial
CCT	Controlled Clinical Trial
PCS	Prospective Case Series
PCT	Prospective Cohort Study
rCS	Retrospective Case Series
rCT	Retrospective Cohort Study
rCC	Retrospective Case–Control
PRISMA	Preferred Reporting Items for Systematic Reviews and Meta-analyses
PROSPERO	International Prospective Register of Systematic Reviews
RoB2	Revised Cochrane Risk-of-Bias Tool for Randomized Trials
NOS	Newcastle Ottawa Scale

## Appendix A

**Table A1:** PICO Guidelines.

Population	Systemically healthy adult patients with alveolar ridge deficiencies.
Intervention	Intraoral Autologous Bone Block Augmentation (ABB).
Comparison	Guided Bone Regeneration (GBR).
Outcome	Patient-Reported Outcomes Measures (PROMs) allowing for a direct comparison of PROMs during Intraoral Bone Block Augmentation procedures and Guided Bone Regeneration.
Study Design	Randomized Controlled Trials, Controlled Clinical Trials (CCT), Prospective Case Series (PCS), Prospective Cohort Studies (PCT), Retrospective Case Series (rCS), Retrospective Cohort Studies (rCT), Retrospective Case–Control (rCC).
Languages	English, Dutch, French, German

**Table A2:** PICO focused search strategy.

	MEDLINE		Embase		Web of Science	
5	#1 AND #2 AND 3 Filters: from 2007–2024	3551	#1 AND #2 AND 3 AND [2007–2024]/py	2423	#1 AND #2 AND #3 and 2024 or 2007 or 2008 or 2009 or 2010 or 2011 or 2023 or 2022 or 2021 or 2020 or 2019 or 2018 or 2016 or 2017 or 2015 or 2014 or 2013 or 2012 (Publication Years)	3705
4	#1 AND #2 AND #3	4555	#1 AND #2 AND 3	2875	#1 AND #2 AND #3	4121
3	((((((((((((postoperative complications[MeSH Major Topic]) OR (intraoperative complications [MeSH Major Topic])) OR (complication)) OR (patient reported outcome)) OR (success)) OR (patient related outcome)) OR (morbidity)) OR (pain)) OR (swelling)) OR (fistula)) OR (hematoma)) OR (sensory)) OR (edema)	12,934,256	‘postoperative complication’/exp OR ‘postoperative complication’ OR ‘peroperative complication’/exp OR ‘peroperative complication’ OR ‘complication’/exp OR ‘complication’ OR ‘patient-reported outcome’/exp OR ‘patient-reported outcome’ OR ‘patient reported outcome measure’/exp OR ‘patient reported outcome measure’ OR ‘success’/exp OR ‘success’ OR ‘patient related outcome’ OR ((‘patient’/exp OR patient) AND related AND (‘outcome’/exp OR outcome)) OR ‘morbidity’/exp OR ‘morbidity’ OR ‘pain’/exp OR ‘pain’ OR ‘pain assessment’/exp OR ‘pain assessment’ OR ‘swelling’/exp OR ‘swelling’ OR ‘fistula’/exp OR ‘fistula’ OR ‘hematoma’/exp OR ‘hematoma’ OR ‘sensory dysfunction’/exp OR ‘sensory dysfunction’ OR ‘sensory’/exp OR ‘sensory’ OR ‘edema’/exp OR ‘edema’	8,422,227	((((((((((((ALL = (postoperative complications)) OR ALL = (intraoperative complications)) OR ALL = (complication)) OR ALL = (patient reported outcome)) OR ALL = (success)) OR ALL = (patient related outcome)) OR ALL = (morbidity)) OR ALL= (pain)) OR ALL = (swelling)) OR ALL = (fistula)) OR ALL = (hematoma)) OR ALL = (sensory)) OR ALL = (edema)	4,264,864
2	(((((((((((Sausage) OR (bone graft)) OR (bone block)) OR (onlay)) OR (alveolar ridge augmentation[MeSH Major Topic])) OR (shell technique)) OR (Khoury)) OR (Split bone block)) OR (bone plate)) OR (guided bone regeneration)) OR (GBR)) OR (Lateral bone augmentation)	15,091	‘sausage’/exp OR sausage OR ‘bone graft’/exp OR ‘bone graft’ OR ‘bone transplantation’/exp OR ‘bone transplantation’ OR ‘bone block’/exp OR ‘bone block’ OR ‘onlay graft’/exp OR ‘onlay graft’ OR ‘onlay technique’/exp OR ‘onlay technique’ OR ‘alveolar ridge augmentation’/exp OR ‘alveolar ridge augmentation’ OR ‘shell technique’ OR ((‘shell’/exp OR shell) AND (‘technique’/exp OR technique)) OR khoury OR ‘split bone block’ OR (split AND (‘bone’/exp OR bone) AND block) OR ‘bone plate’/exp OR ‘bone plate’ OR ‘guided bone regeneration’/exp OR ‘guided bone regeneration’ OR gbr OR ‘lateral bone augmentation’ OR (lateral AND (‘bone’/exp OR bone) AND (‘augmentation’/exp OR augmentation))	278,771	(((((((((((ALL = (sausage)) OR ALL = (bone graft)) OR ALL = (bone block)) OR ALL= (onlay)) OR ALL = (alveolar ridge Augmentation)) OR ALL = (shell technique)) OR ALL= (khoury)) OR ALL = (split bone block)) OR ALL = (bone plate)) OR ALL = (guided bone regeneration)) OR ALL = (GBR)) OR ALL = (Lateral bone augmentation)	248,670
1	Dental Implants	52,994	‘tooth implantation’/exp OR ‘tooth implantation’ OR ‘tooth implant’/exp OR ‘tooth implant’	48,286	ALL = (dental implants)	65,802
