# Supplementary material for: Patient-Reported Outcomes in Intraoral Bone Block Augmentation Compared to GBR Procedures Prior to Implant Placement: A Systematic Review
Source: J Clin Med. 2025 Jul 28;14(15):5331. doi: 10.3390/jcm14155331 (PMC12347253; doi:10.3390/jcm14155331)
Supplement: Supplementary file 1 [file jcm-14-05331-s001.zip › jcm-3725292-supplementary/Supplementary Files/Supplementary Table S1.pdf]

Supplementary Table S1: Reasons for exclusion

Sinus Augmentation	Irrelevant to the main topic	No reporting of PROMs	No usage of autologous bone
Urban IA et al., 2014 [1]	SM Meloni et al., 2019 [2]	D Schwartz-Arad et al., 2016 [3]	Vrisiis Kofina et al., 2023 [4]
Francesco Pieri et al., 2008 [5]		A Bartols et al., 2018 [6]	
Urban IA et al., 2013 [7]			
Urban IA et al., 2011[8]			

References

1. Urban, I.A.; Lozada, J.L.; Jovanovic, S.A.; Nagursky, H.; Nagy, K. Vertical ridge augmentation with titanium-reinforced, dense-PTFE membranes and a combination of particulated autogenous bone and anorganic bovine bone-derived mineral: a prospective case series in 19 patients. Int J Oral Maxillofac Implants 2014, 29, 185-193, doi:10.11607/jomi.3346.

2. Meloni, S.M.; Jovanovic, S.A.; Urban, I.; Baldoni, E.; Pisano, M.; Tallarico, M. Horizontal ridge augmentation using GBR with a native collagen membrane and 1:1 ratio of particulate xenograft and autologous bone: A 3-year after final loading prospective clinical study. Clin Implant Dent Relat Res 2019, 21, 669-677, doi:10.1111/cid.12808.

3. Schwartz-Arad, D.; Ofec, R.; Eliyahu, G.; Ruban, A.; Sterer, N. Long Term Follow-Up of Dental Implants Placed in Autologous Onlay Bone Graft. Clin Implant Dent Relat Res 2016, 18, 449-461, doi:10.1111/cid.12288.

4. Kofina, V.; Monfaredzadeh, M.; Rawal, S.Y.; Dentino, A.R.; Singh, M.; Tatakis, D.N. Patient-reported outcomes following guided bone regeneration: Correlation with clinical parameters. J Dent 2023, 136, 104605, doi:10.1016/j.jdent.2023.104605.

5. Pieri, F.; Corinaldesi, G.; Fini, M.; Aldini, N.N.; Giardino, R.; Marchetti, C. Alveolar ridge augmentation with titanium mesh and a combination of autogenous bone and anorganic bovine bone: a 2-year prospective study. J Periodontol 2008, 79, 2093-2103, doi:10.1902/jop.2008.080061.

6. Bartols, A.; Kasprzyk, S.; Walther, W.; Korsch, M. Lateral alveolar ridge augmentation with autogenous block grafts fixed at a distance versus resorbable Poly-D-L-Lactide foil fixed at a distance: A single-blind, randomized, controlled trial. Clin Oral Implants Res 2018, 29, 843-854, doi:10.1111/clr.13303.

7. Urban, I.A.; Nagursky, H.; Lozada, J.L.; Nagy, K. Horizontal ridge augmentation with a collagen membrane and a combination of particulated autogenous bone and anorganic bovine bone-derived mineral: a prospective case series in 25 patients. Int J Periodontics Restorative Dent 2013, 33, 299-307, doi:10.11607/prd.1407.

8. Urban, I.; Nagursky, H.; Lozada, J. Horizontal Ridge Augmentation with a Resorbable Membrane and Particulated Autogenous Bone With or Without Anorganic Bovine Bone-Derived Mineral: A Prospective Case Series in 22 Patients. The International journal of oral & maxillofacial implants 2011, 26, 404-414.
