# Supplementary material for: Patient-Reported Outcomes in Intraoral Bone Block Augmentation Compared to GBR Procedures Prior to Implant Placement: A Systematic Review
Source: J Clin Med. 2025 Jul 28;14(15):5331. doi: 10.3390/jcm14155331 (PMC12347253; doi:10.3390/jcm14155331)
Supplement: Supplementary file 1 [file jcm-14-05331-s001.zip › jcm-3725292-supplementary/Supplementary Files/Supplementary Table S2.pdf]

Supplementary Table S2: Instruments used for reporting PROMs

Author	PROM instrument	Follow-up schedule	Outcome Variables
Bayram et al., 2024 [1]	Clinical records<br>Reporting by patients verbally	Week 1<br>1 year	Neurosensory disturbances
Shiezadeh et al., 2023 [2]	Reporting by patients verbally	26 Weeks	Neurosensory disturbances in donor sites (ABB only) and recipient sites (ABB and GBR)<br>swelling
Thoma et al., 2019 [3]	Questionnaires	Mean follow-up of 10 years	Sensory problems<br>Willingness to repeat the procedure<br>Likelihood to recommend the procedure
Lorenz et al., 2025 [4]	VAS-score<br>OHIP-14 questionnaire	Pretreatment<br>Weeks 1,3,12 and 24<br>Time of implant placement<br>Definitive prosthesis<br>6 months<br>8 months<br>1 year	Pain<br>OHIP-14
Sakkas et al., 2016 [2]	Clincal records<br>Reporting by patients verbally	NS	Neurosensory disturbance
Korsch et al., 2014 [5]	VAS-score<br>Reporting by patients verbally	Day 1, 3, 7, 14 and 28	Pain and swelling in the donor and recipient sites,<br>Postoperative analgesic usage<br>Patient-Reported Predominant Symptom

NS: Not-Specified

References

1. Bayram, F.; Göçmen, G.; Özkan, Y. Evaluating risk factors and complications in mandibular ramus block grafting: a retrospective cohort study. Clin Oral Investig 2024, 28, 226, doi:10.1007/s00784-024-05613-6.
2. Sakkas, A.; Ioannis, K.; Winter, K.; Schramm, A.; Wilde, F. Clinical results of autologous bone augmentation harvested from the mandibular ramus prior to implant placement. An analysis of 104 cases. GMS Interdiscip Plast Reconstr Surg DGPW 2016, 5, Doc21, doi:10.3205/iprs000100.
3. Thoma, D.S.; Maggetti, I.; Waller, T.; Hämmerle, C.H.F.; Jung, R.E. Clinical and patient-reported outcomes of implants placed in autogenous bone grafts and implants placed in native bone: A case-control study with a follow-up of 5-16 years. Clin Oral Implants Res 2019, 30, 242-251, doi:10.1111/clr.13410.
4. Lorenz, J.; Ghanaati, S.; Aleksic, Z.; Milinkovic, I.; Lazic, Z.; Magić, M.; Wessing, B.; Grotenclos, R.S.; Merli, M.; Mariotti, G.; et al. Horizontal Guided Bone Regeneration of the Posterior Mandible to Allow Implant Placement: 1-Year Prospective Study Results. Clin Oral Implants Res 2025, 36, 100-116, doi:10.1111/clr.14363.
5. Korsch, M.; Robra, B.-P.; Kasprzyk, S.; Walther, W. Patients' perception of postoperative discomfort after bone graft: A parallel randomized clinical study of two techniques for lateral bone graft. Implantologie 2014, 22, 379-388.
